# A New Patient-Derived Metastatic Glioblastoma Cell Line: Characterisation and Response to Sodium Selenite Anticancer Agent

**DOI:** 10.3390/cancers11010012

**Published:** 2018-12-21

**Authors:** Sylvie Berthier, Louis Larrouquère, Pierre Champelovier, Edwige Col, Christine Lefebvre, Cécile Cottet-Rouselle, Josiane Arnaud, Catherine Garrel, François Laporte, Jean Boutonnat, Patrice Faure, Florence Hazane-Puch

**Affiliations:** 1 Cytometry Platform, Institute of Biology and Pathology, Grenoble Alpes Hospital, CS10217, Grenoble CEDEX 9, France; SBerthier@chu-grenoble.fr (S.B.); pierre.champelovier@wanadoo.fr (P.C.); 2 BrainTech Lab, INSERM U1205, 38000 Grenoble, France; LLarrouquere@chu-grenoble.fr; 3 Medical Oncology Department, Grenoble Alpes Hospital, CS10217, Grenoble CEDEX 9, France; 4 Unit of Anatomopathology, Institute of Biology and Pathology, Grenoble Alpes Hospital, CS10217, Grenoble CEDEX 9, France; ECol@chu-grenoble.fr (E.C.); JBoutonnat@chu-grenoble.fr (J.B.); 5 Laboratory of Hematology, Onco-Genetic and Immunology, Institute of Biology and Pathology, Grenoble Alpes Hospital, CS10217, Grenoble CEDEX 9, France; CLefebvre@chu-grenoble.fr; 6 Laboratory of Fundamental and Applied Bioenergetics (LBFA) and SFR BEeSy, University Grenoble Alpes, Inserm U1055, 38000 Grenoble, France; cecile.cottet@univ-grenoble-alpes.fr (C.C.-R.); JArnaud@chu-grenoble.fr (J.A.); 7 Unit Nutritional and Hormonal Biochemistry, Institute of Biology and Pathology, Grenoble Alpes Hospital, CS10217, 38043 Grenoble CEDEX 9, France; J.A, CGarrel@chu-grenoble.fr (C.G.); francois.laporte@cegetel.net (F.L.); PFaure@chu-grenoble.fr (P.F.); 8 Hypoxia-Physiopathology Laboratory (HP2), Inserm U1042, University Grenoble Alpes, 38000 Grenoble, France

**Keywords:** Glioblastoma, cancer stem cells, new cell line, sodium selenite, xenograft, cell death, epigenetics

## Abstract

Glioblastoma multiform (GBM) tumors are very heterogeneous, organized in a hierarchical pattern, including cancer stem cells (CSC), and are responsible for development, maintenance, and cancer relapse. Therefore, it is relevant to establish new GBM cell lines with CSC characteristics to develop new treatments. A new human GBM cell line, named R2J, was established from the cerebro-spinal fluid (CSF) of a patient affected by GBM with leptomeningeal metastasis. R2J cells exhibits an abnormal karyotype and form self-renewable spheres in a serum-free medium. Original tumor, R2J, cultured in monolayer (2D) and in spheres showed a persistence expression of CD44, CD56 (except in monolayer), EGFR, Ki67, Nestin, and vimentin. The R2J cell line is tumorigenic and possesses CSC properties. We tested in vitro the anticancer effects of sodium selenite (SS) compared to temozolomide TMZ. SS was absorbed by R2J cells, was cytotoxic, induced an oxidative stress, and arrested cell growth in G2M before inducing both necrosis and apoptosis via caspase-3. SS also modified dimethyl-histone-3-lysine-9 (H3K9m2) levels and decreased histone deacetylase (HDAC) activity, suggesting anti-invasiveness potential. This study highlights the value of this new GBM cell line for preclinical modeling of clinically relevant, patient specific GBM and opens a therapeutic window to test SS to target resistant and recurrent GBM.

## 1. Introduction

Glioblastoma represents the most common type of primary tumors of the central nervous system (CNS) and has a poor prognosis [1]. Recent studies have clarified the common somatic genetic alterations that occur in human GBM. The cellular signaling pathways, including phosphatidylinositol-3 kinase (PI3K) and cell cycle deregulation, are often related to the malignant transformation [2]. Indeed, cyclin-dependent kinase inhibitors, including p15/INK4b, p16/INK4A, p21/Waf1, p27/Kip1, and Mouse double minute 2 homolog (MDM2), play a primordial role in this process [3]. Specific alterations affecting these pathways that include aberrant expression of oncogenes and tumor suppressor genes are shown to be correlated to tumor prognosis, disease progression, and cancer metastasis [4]. Among them, amplification of several growth factor receptors, including EGFR, have been associated to a poor prognosis in GBM [5]. Moreover, Ceccarelli et al. classified GBM in different subtypes, especially dependent on their transcriptional signature and with a correlation with therapeutic response [6].

Recent findings have identified a subpopulation of stem-like cells within tumors, known as cancer stem cells (CSC), exhibiting characteristics of both stem cells and cancer cells. In addition to self-renewal and differentiation capacities, CSCs have the ability to seed tumors when transplanted into the animal host [7,8]. Concerning the origin of CSCs, one hypothesis is that these cells arise from normal somatic cells acquiring stem-like characteristics and malignant behavior, for example, via a glial to mesenchymal transition (GMT) [8]. CSCs are highly resistant to current therapies and could be responsible for repopulating the initial GBM tumor, which explain the high recurrence of tumors [7]. To date, there is no single marker for CSCs, rather a combination of different markers is used to identify CSCs in an isolated cell line. Indeed, the expression of Nestin and Sox-2 has been associated with both the GMT process and CSCs [8,9,10]. CD34 and CD133 [11] have been used to isolate CSCs from different tumors [12]. The GMT, a crucial process in glial tumor progression, is characterized by the reduction of glial markers (GFAP, which is also used as an astrocytic lineage-specific marker [13]) in exchange for increased expression of mesenchymal markers, such as vimentin [14]. Moreover, the expression of several markers, including Snail, Sox-2, EGFR, CD44, and Six-1, has been associated with the GMT process [10,15]. It should be noted that the induction of epithelial (E)-MT also significantly led to the expression of markers associated with breast CSCs (for a review see [16]). In GBM, the malignant cells are thought to acquire motility by degrading or interacting with extracellular matrix (ECM) components through matrix metalloproteases (MMPs) and specific cell surface molecules, respectively. In this process, CD44, highly expressed during GMT [17], plays an important role in the implantation of tumor cells, allowing the initiation of a metastatic cascade related to the modulation of several cellular characteristics, including adhesion, motility, and matrix degradation [18]. Epithelial markers include adherens and tight junction proteins, such as E-cadherin and ZO-1, whereas mesenchymal markers include, for example, the extracellular matrix component, fibronectin, and the intermediate filament protein, vimentin (for a review see [17]).

Epigenetic modifications are implied in GBM progression [19]. Histone (H) methylation is a complex process implying several histone lysine demethylases (KDM) associated to a variety of physiological and pathological conditions, including cancer. Histone methylation involves specific lysine and arginine of H3 and H4 implied in the regulation of gene transcription and in the support of gene maintenance either on activation or on a repressive dynamic process [19]. Lower global levels of H3K9m2 predict poor prognosis in kidney and prostate cancers [20], but not in lung cancer [21]. Concerning H3K9m2 level regulation, some KDM has been studied, such as LSD1 (KDM1), associated with demethylation of H3K9m1/m2, lysine-specific demethylase 4C (KDM4C/JMJD2C), and G9a, which demethylase H3K9m2/m3 [22].

Temozolomide (TMZ) is the standard of care for GBM, but it still has limitations and unsatisfactory outcomes, particularly in patients with tumor cells expressing the O(6)-methyguanine-DNA-methyltransferase (MGMT) [1,23]. Moreover, resistance to TMZ has become a major concern in GBM treatment, thus new therapies are urgently needed.

In this way, we study sodium selenite’s (SS) anticancer properties. We have previously showed that the chemical form and doses influenced its ways of actions [24,25] and we investigated SS anticancer properties in human GBM cell lines [26,27]. We, and others, indicated that SS induces cell death via oxidative stress. Indeed, in the T98G GBM cell line, 24h SS (5, 10 µM) treatment induced a significant decrease of thiol groups and glutathione [27].

The susceptibility of the cultures to the effects of SS depends on the cell type. Indeed, several authors showed that SS was preferentially toxic to malignant glioma cells over normal astrocytes. For example, Rooprai et al. [28] found an IC50 of 28.9 µM for SS in anaplastic astrocytoma within 8 h of treatment whereas it was not reached for 69.36 µM in normal human astrocytes. Kim et al. [29] reported an IC50 comprised between 3 and 5 µM for the established human GBM cell lines (U87MG, U343, A172, and U251) treated during 24 h, whereas, interestingly, no toxicity was reported with 7 µM of SS in normal astrocytes [29]. In vivo, SS accumulates preferentially in the tumor rather than the normal brain tissue in Wistar rats bearing C6 glioma and treated for 4 weeks with 2 ppm to 5 ppm of SS in drinking water [30].

This last point is of great relevance for GBM therapy as crossing the blood brain barrier (BBB) is one of the challenge of new drugs: SS crosses the BBB [31].

Anti-cancer properties of sodium selenite in vitro are so encouraging that SS deserves to be deeply studied for chemotherapeutic responses, before being given up. The clinical trial, SECAR (Sodium Selenite as a Cytotoxic Agent in Advanced Carcinoma, https://clinicaltrials.gov/ct2/show/study/NCT01959438), is ongoing. The phase I has allowed determination of the maximal tolerated dose (MTD) as 10.2 mg/m² (phase I). The aim of the phase II, which is in progress, is to use MTD and to study responses, if any, in malignant tumor and in treatment resistant tumors.

The aim of our study was first to characterize this new cell line, named R2J, which expresses the MGMT transcript and exhibits CSC properties associated with in vivo tumor growth, and, second, to evaluate in vitro SS effects.

## 2. Results

### 2.1. Immunohistochemistry of the Original Tumor

**Figure 1:**
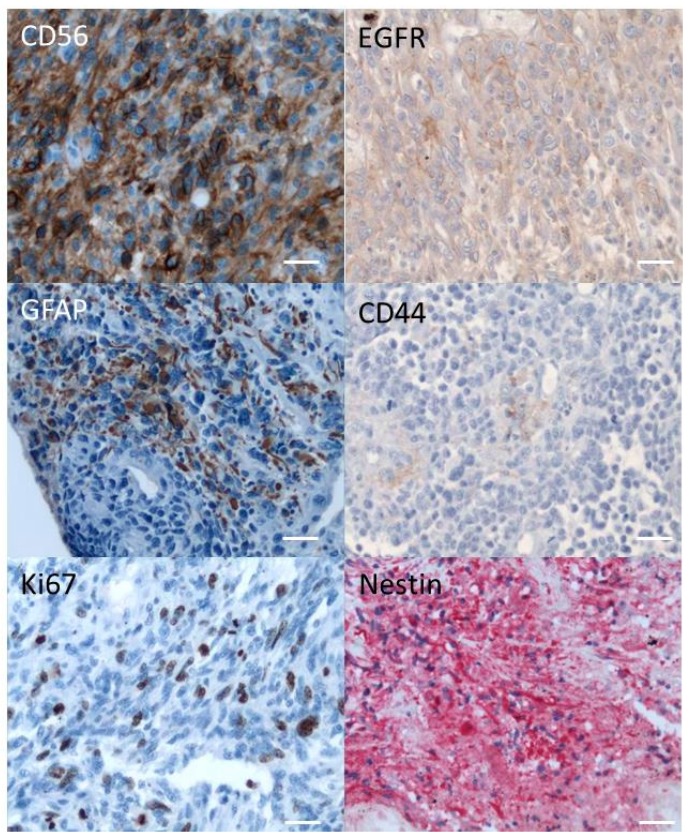
Original tumor was labelled with different markers (see Table 1) as described in the Materials and Methods, using a revelation DAB (peroxidase) for CD56, CD44, GFAP, EGFR, and Ki67 and a revelation Fast Red (alcaline phosphatase) for Nestin. Only positive labellings are shown. Pictures were captured using Leica ICC50 camera connected to a Leica DM2500 microscope (objective ×20). Scale bar: 100 µm.

The resected tumor was positive for GFAP, Ki67, vimentin, CD56, and nestin. Only few cells were positive for EGFR and CD44 (Figure 1, Table 1). It is worth noting that Ki67 staining can reach 30% on some focus. Neuronal markers, NF70, synaptophysin, and NeuN, were also tested and were not detected.

### 2.2. Phenotypic Characterisation of Tumor and R2J Cells

**Figure 2:**
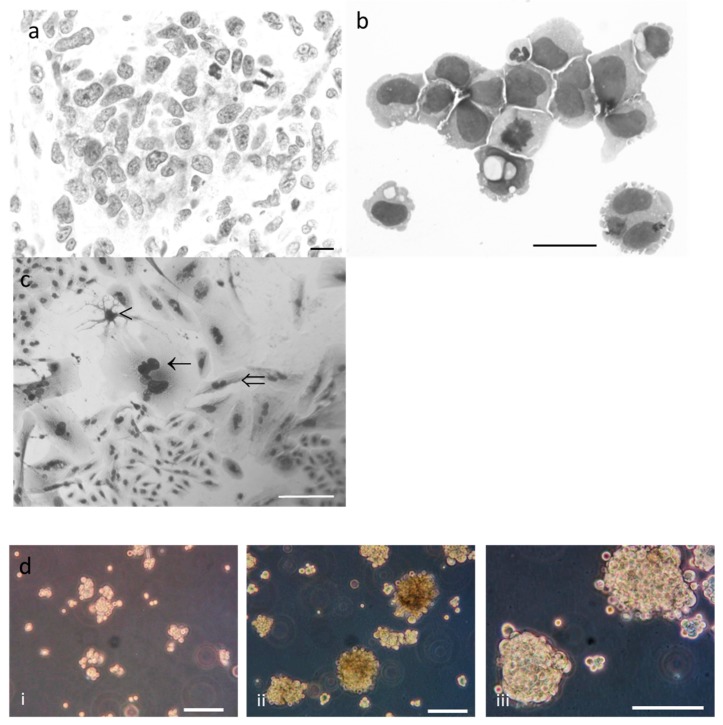
Morphological and phenotypic analysis of (**a**) the biopsy of the original tumor: HES staining shows the heterogeneity of the tumor cells and mitosis. Scale bar = 100 µm (**b**) cerebro-spinal fluid (CSF): May Grunwald Giemsa (MGG) staining shows mitosis and a giant cell. Scale bar = 25 µm (**c**) R2J cells in 2D culture: MGG staining shows giant cells (←), fibroblastic-like cells (⇐) and glial-like cells (<). Scale bar = 100 µm (d) R2J forming spheres in a medium without serum, 7 days (**i**) and 25 days (**ii**: ×100 and iii: ×200) after the seeding. Pictures are representative of more than four independent experiments. Scale bar = 100 µm.

Original tumor cells were heterogeneous with a big nucleolus (Figure 2a). Numerous mitosis was shown with a high mitotic index (MI) > 5%. More than 90% of the cells from CSF were round and uniform (10–15 µm) (Figure 2b); the nucleus was round with numerous nucleoli. Most of mitotic figures appeared morphologically normal (MI = 2%). Less than 5% of the cells were multinucleated.

Currently, R2J cells are maintained under passage 50 and retained cellular phenotypes’ diversity. Indeed, more than 70% of the cells were round and small (15–20 µm), 10% of the cells were round, giant (30–50 µm), and multinucleated (2–5 nuclei), and less than 20% of the cells exhibited fibroblastic- or glial-like morphology (Figure 2c). Cytospin slides stained with MGG essentially showed small and giant cells.

R2J cells were able to form colonies with a PE = 12.4% ± 3.1 (PE = (counted colonies/cells plated) × 100).

### 2.3. R2J Cells are Able to Form Gliospheres

When R2J are plated in a cell repellent 10 cm-dish, small spheres containing 10–20 cells were formed 5–6 days after the plating. The sphere diameter increased each day and tumor spheres can be passaged every week for many generations in fresh medium without serum (Figure 2d). The percentage of TS-ICs to examine the efficacy of clonogenesis was 1.9% ± 0.1 (*n* = 3 independent experiments).

### 2.4. Immunohistochemistry of R2J Cells Cultured in 2D and in Gliospheres

**Figure 3:**
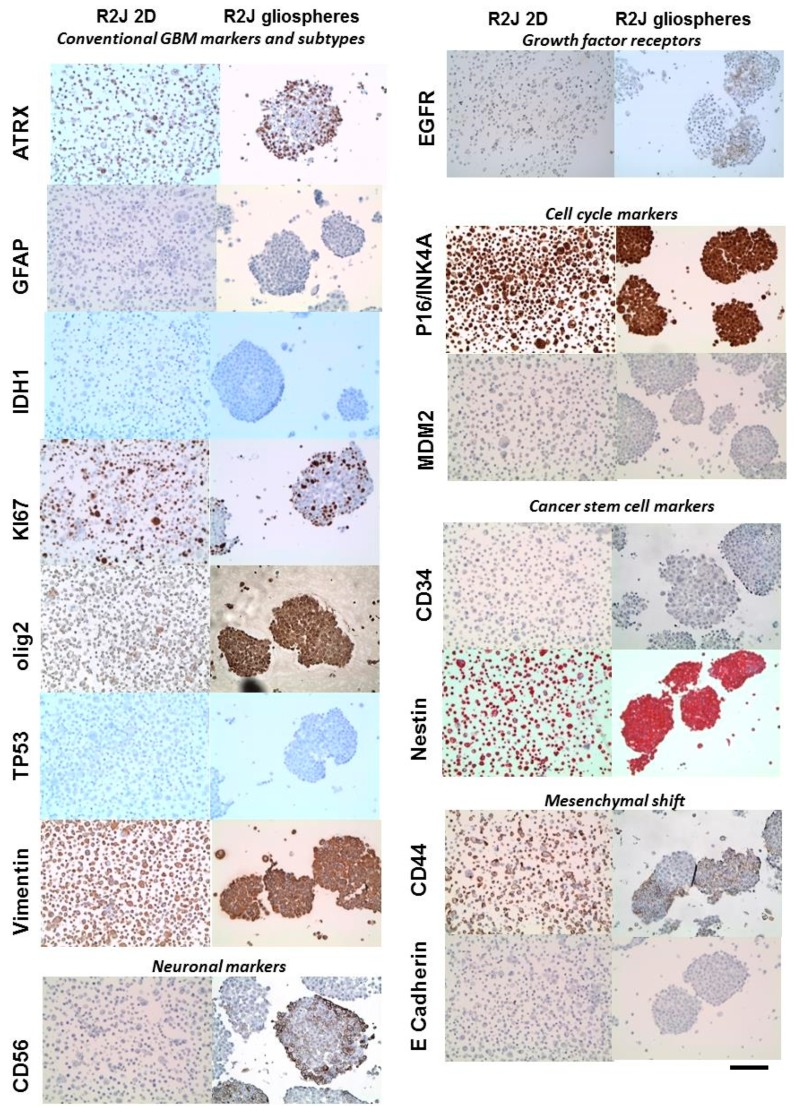
R2J cells cultured in monolayer or in spheres were labelled with different markers as described in the Materials and Methods. Scale bar = 100 µM.

Compared to the original tumor, R2J cells in culture (2D and spheres) lost the GFAP and CD56 expressions (only 2D) whereas Ki67, vimentin and nestin expressions were conserved as well as mesenchymal shift markers, such as CD44 (Figure 3, Table 1).

Comparing 2D vs. spheres, it appears that only olig2 and CD56 were expressed in spheres. E-Cad transcript was tardily detected in RT-q-PCR (Ct = 37.1 ± 0.9) and the protein was not detected (Figure 3). Concerning Sox2 transcript, it was detected early by RT-q-PCR both in 2D and spheres cells (Ct = 21.4 ± 0.9 and 24.5 ± 2.3, respectively). Moreover, N-Cad transcript was neither detected in adherent R2J cells nor in spheres.

### 2.5. MGMT Status of R2J Cells

R2J cells expressed MGMT transcript (evaluated by RT-q-PCR) with a cycle threshold (Ct) value=34.8 ± 4.1 (*n* = three independent experiments). U251 cell line was used as a negative control for MGMT status (no Ct) and T98G was used as a positive control with Ct = 26.11 ± 0.04 (*n* = three independent experiments).

### 2.6. Chromosome Analysis

Karyotype analysis, at passages 5 and 35, showed that proliferative R2J cells possess an abnormal karyotype (Supplementary Material, Figure S1). R2J cells are hypotriploid (modal number 64) and showed a large number of numerical abnormalities: A recurrent loss of chromosomes (chr-) 6, 8, 9, 10, 11, 13, 21, 22, and X, a gain of chr-7 (five copies), chr-14 (four copies), and chr-19 (four copies). The chr-Y was not observed whereas R2J was from a male patient. One recurrent structural change (add 7q11) was always present. This was consistent with the degree of malignancy of the original tumor (diagnosed GBM). Moreover, analysis of DNA content by flow cytometry confirmed the polyploidy of the R2J cells.

### 2.7. R2J Cells are Tumorigenic and Cancer Stem Cells

**Figure 4:**
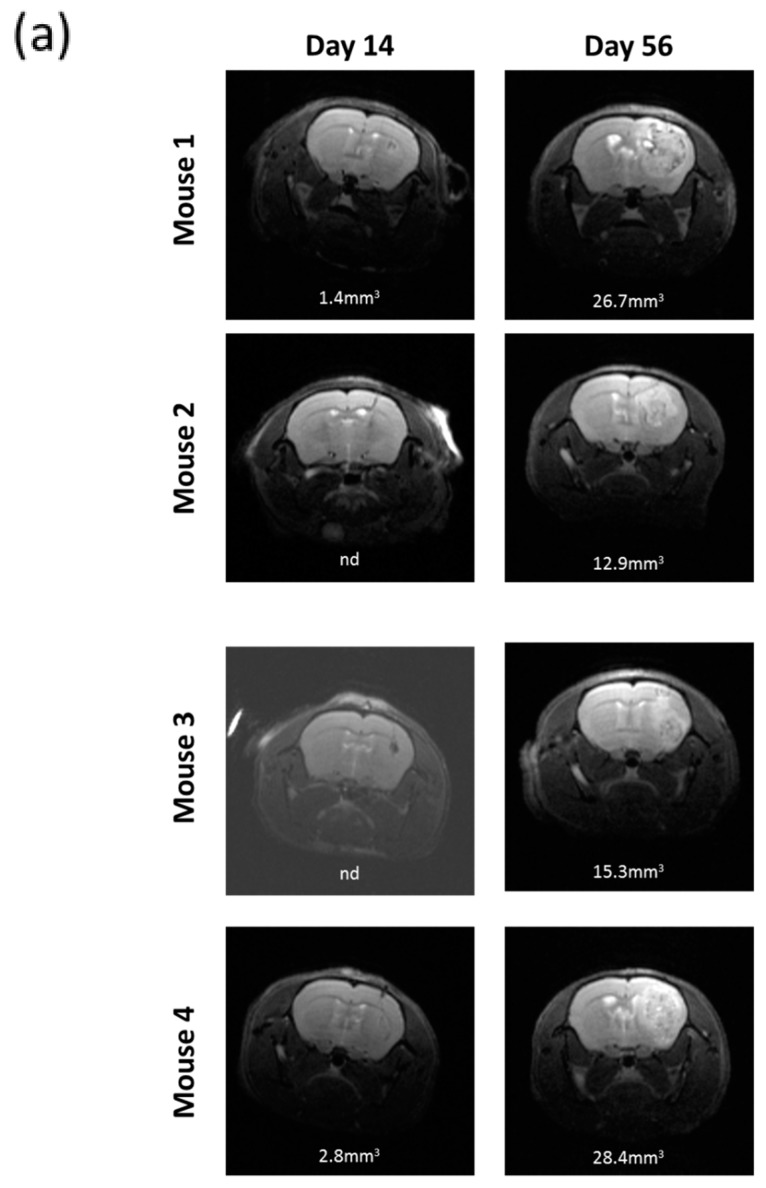

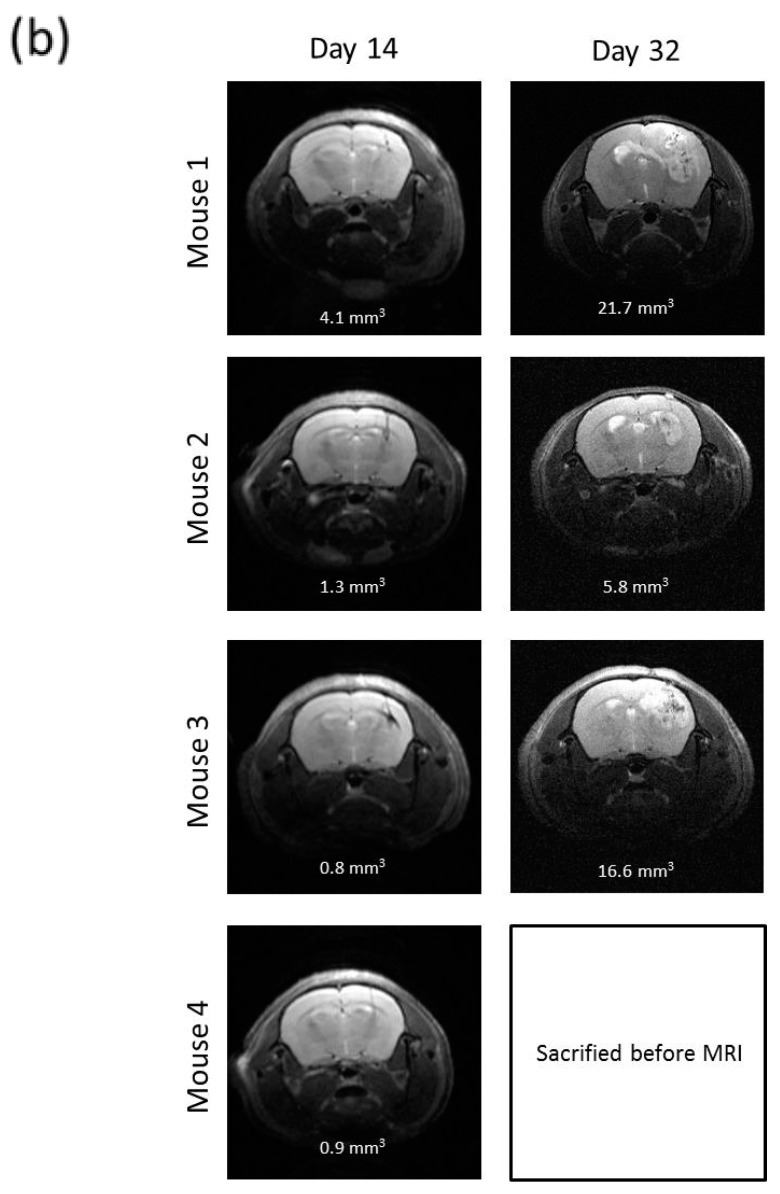

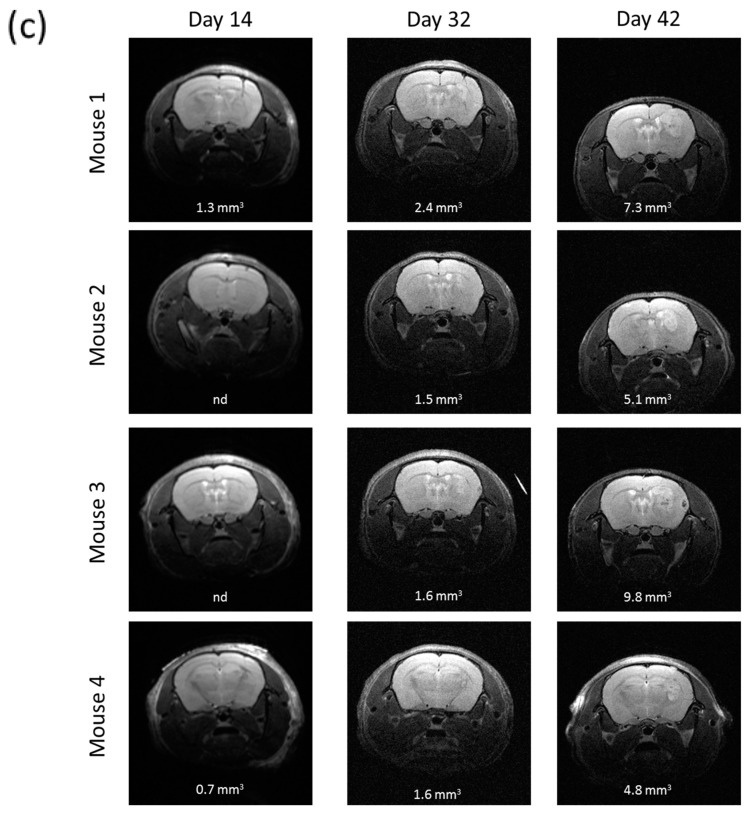
In vivo tumorigenicity of R2J cells after intracranial implantation in nude mice of (**a**) 2 × 10^5^ cells cultivated in the monolayer (**b**) 2 × 10^5^ or (**c**) 1000 cells cultivated in spheres. MRI acquisitions were performed post implantation at the times indicated. Mice were sacrificed after the last MRI. Tumor volumes were calculated by adding each tumor x slice thickness (0.5 mm²). (**a**) Implantation of 2 × 10^5^ R2J monolayer cultivated cells. (**b**) Implantation of 2 × 10^5^ R2J sphere cells. (**c**) Implantation of 1000 R2J sphere cells.

All the nude mice intracranially implanted with R2J cells cultivated in monolayer (2 × 10^5^ cells, *n* = 4) and in spheres (2 × 10^5^ cells, *n* = 4 and 1000 cells, *n* = 4) were tumor bearing (Figure 4). Two weeks after the implantations, MRI revealed the presence of tumors in mice, which was confirmed 56 days post implantation (PI) for monolayer cells (Figure 4a) and 32 days PI for spheres (Figure 4b,c).

### 2.8. SS Absorption

Se was measured by Inductively Coupled Plasma Mass Spectrometry ICP-MS both in lysates and medium in R2J-2D cells treated with SS. The quantity of Se absorbed significantly increased with the SS concentration added. Indeed, at 2.5 µM, the percentage of Se measured vs. Se added was 0.6% ± 0.2 vs. 2.8% ± 0.7 at 5 µM (*p* < 0.05 vs. 2.5 µM) vs. 3.7% ± 1.3 at 10 µM (*p* < 0.005 vs. 2.5 µM) for *n* = three independent experiments.

Se recovery was 102.7% ± 1.1 at 2.5 µM vs. 83.2%±4.1 at 5 µM (*p* < 0.0001 vs. 2.5 µM) and vs. 79.9% ± 7.0 at 10 µM (*p* < 0.0001 vs. 2.5 µM) for *n* = three independent experiments. It means that a loss of Se depended on the concentration added.

### 2.9. SS Triggered both Apoptosis and the Necrosis Cell Death Process

**Figure 5:**
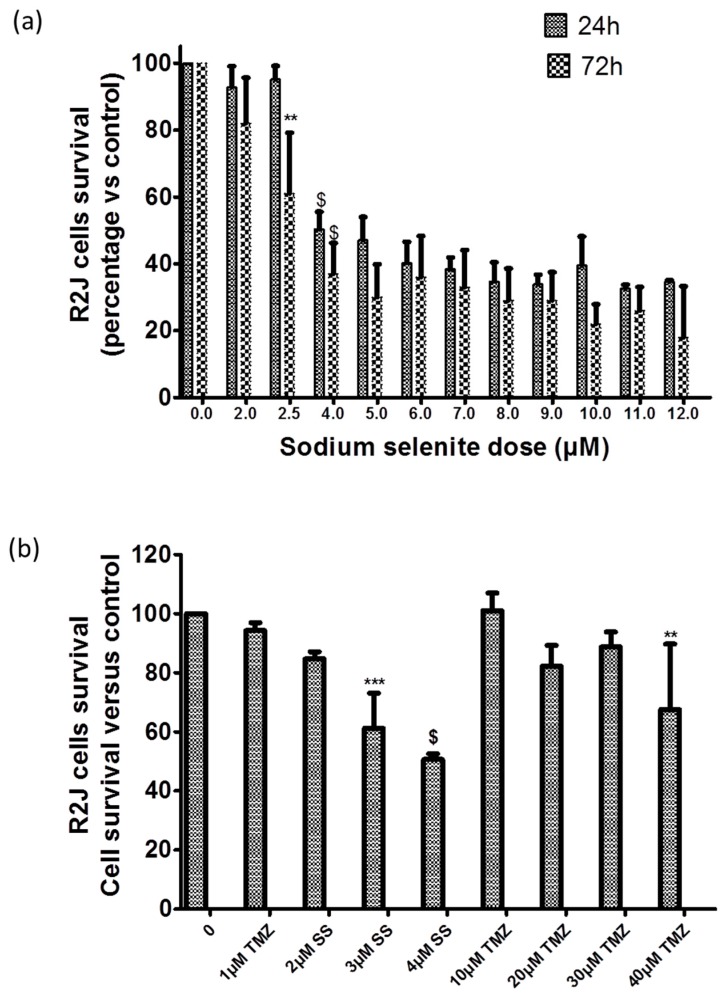

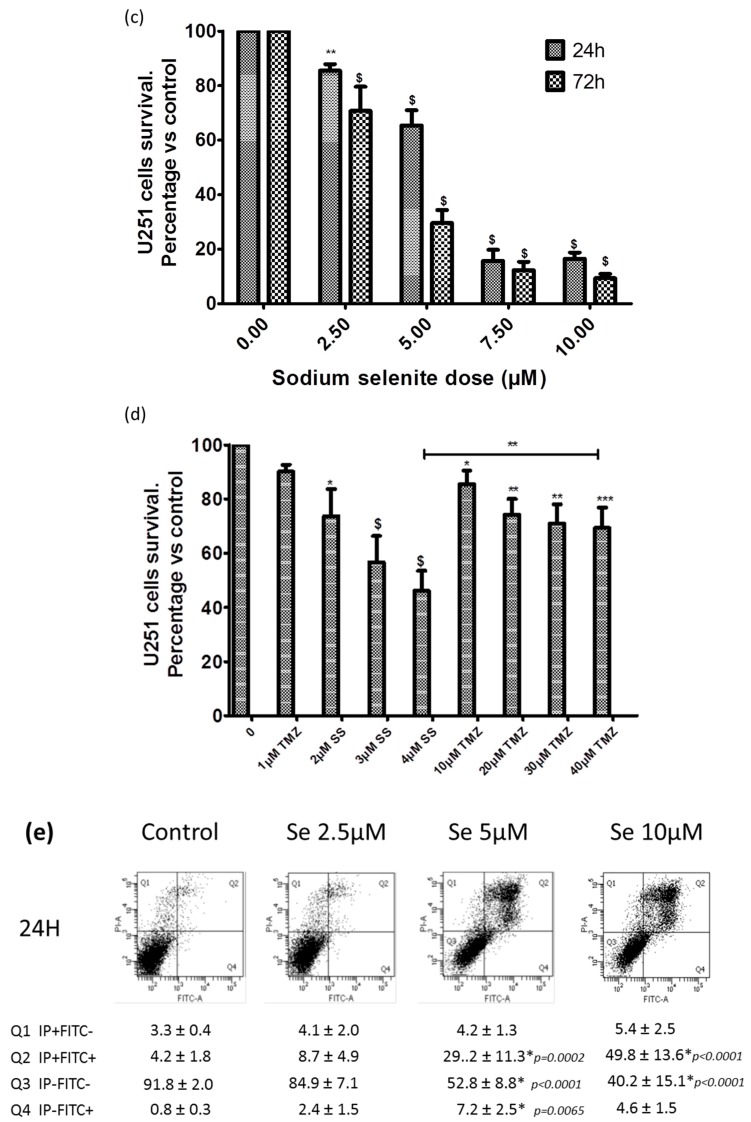

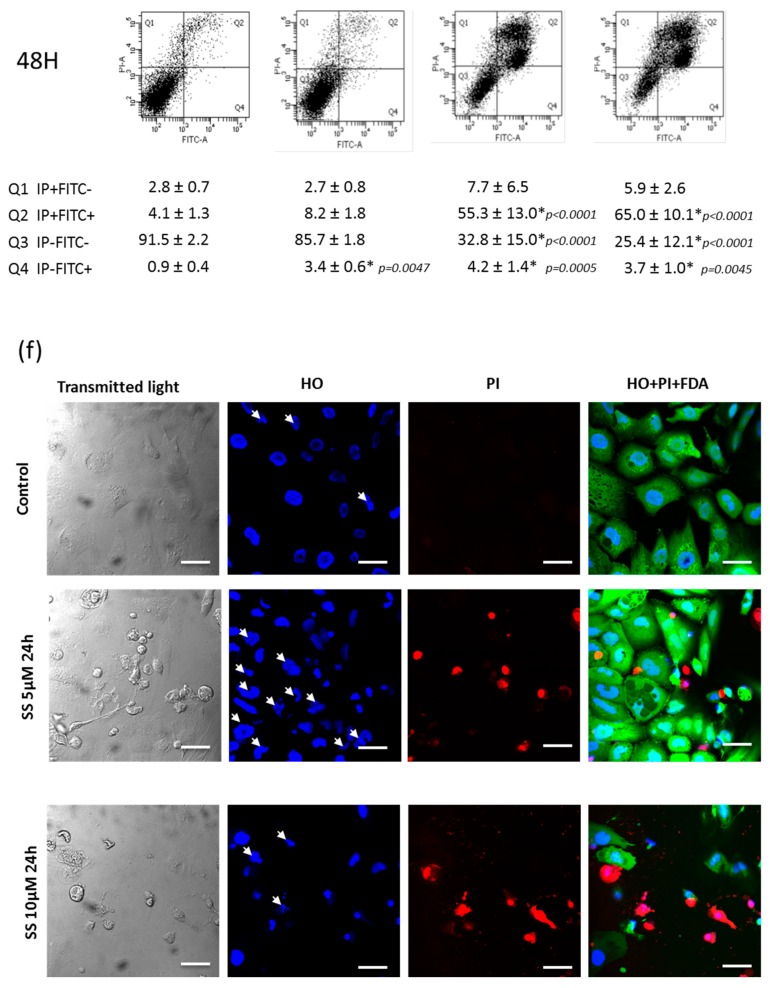

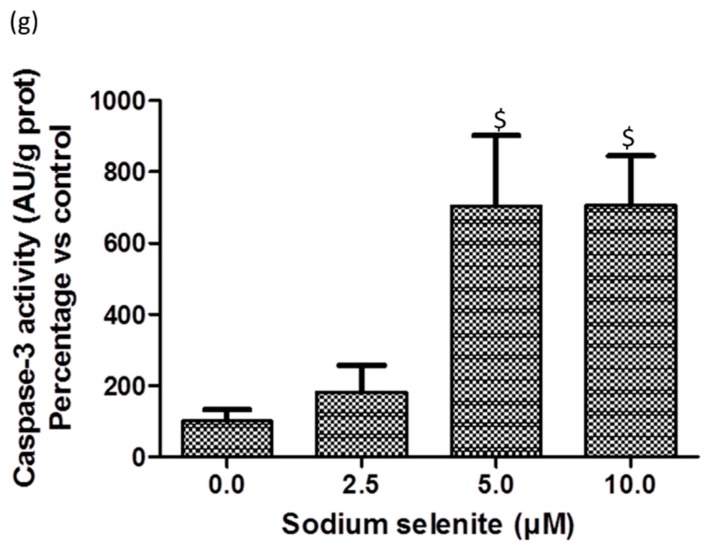
Sodium selenite (SS) cytotoxicity and cell death triggered. SS is more cytotoxic than TMZ in R2J ((**a**) and (**b**)) and in U251 ((**c**) and (**d**)) cells: Cell survival was evaluated by an MTT assay performed on cell growing without (control = 100%) or with variable doses of sodium selenite for 24 h and 72 h (**a**,**c**) or sodium selenite or TMZ for 72 h, followed by 72 h of wash-out (**b**,**d**). Results, expressed in percentage of cell survival vs. the control, are the mean ± SD of three independent experiments with * *p* < 0.05, ** *p* < 0.005, *** *p* < 0.0005, and ^$^ *p* < 0.0001 vs. control. In (**a**), only the first statistical significance was specified to avoid an overloading of the graph. (**e**) Apoptosis (Annexin-V labelling) and necrosis (PI labelling) were evaluated by flow cytometry analysis, 24 h and 48 h after SS treatment. Results are the mean ± SD of four independent experiments. The percentage of cells in each quadrant was compared as a function of the SS concentration and when significant, the p value was informed. Q3 represents viable cells, Q1: Necrotic cells, Q2: Both necrotic and apoptotic cells, and Q4: Apoptotic cells. (**f**) Confocal microscopy: R2J were exposed to different concentrations of sodium selenite for 24 h and labelling was compared to the controls. Intact cells were revealed by intracellular green fluorescence of FDA (no loss of plasma membrane integrity) whereas the nuclei of necrotic cells were labelled with propidium iodide (PI) (red fluorescence). The Hoechst blue fluorescence allowed the discrimination of apoptotic nuclei with an irregular shape (informed white arrows) that exhibited bean-like morphology or were fragmented whereas the intact nuclei displayed a regular shape. Pictures are representative of three independent experiments. Scale Bar: 40 µm. (**g**) SS triggered apoptosis via caspase-3. Caspase 3 assay was performed in R2J cells seeded at 6000 cells/cm² and treated with SS for 24 h. Cells and medium were then recovered, centrifuged for 3 min, 360 g, room temperature and rinsed twice with PBS. Cells were lysed in 50 µL of the Caspase-3 lysis buffer. Results are mean ± SD of three independent experiments with ^$^ *p* < 0.0001 versus the control.

For SS treatment in R2J-2D cells, IC50 values were 3.4 µM ± 0.2 and 2.6 µM ± 0.2 at 24 h and 72 h, respectively (Figure 5a), whereas IC50 was not reached after TMZ acute treatment, until 1mM for 72 h [32] or after the long term treatment, although TMZ was significantly cytotoxic at 40 µM (Figure 5b). Our results suggest that R2J cells are resistant to TMZ. It should be noted that upper TMZ doses were not testable due to DMSO cytotoxicity. U251, which are MGMT negative and p53 mutant, exhibited comparable IC50, i.e., 5.44 µM ± 0.02 and 3.50 µM ± 0.03 at 24 h and 72 h, respectively (Figure 5c), but were more sensitive to the TMZ long term treatment with a significant cell mortality at 10 µM (Figure 5d).

Flow cytometry analysis showed that SS significantly triggered both necrosis and apoptosis at 5 µM for 24 h which was amplified at 48 h (Figure 5e). These results were confirmed by confocal analysis with increased PI staining, a nuclear bean shape specific of apoptosis, and loss of fluorescein diacetate (FDA) at 5 µM-24 h (Figure 5f).

The induction of apoptosis by SS was finally confirmed by a significant increase of caspase-3 activity at 5 µM-24 h (Figure 5g).

### 2.10. SS Induced DNA Fragmentation and Cell Cycle Arrest in G2

**Figure 6:**
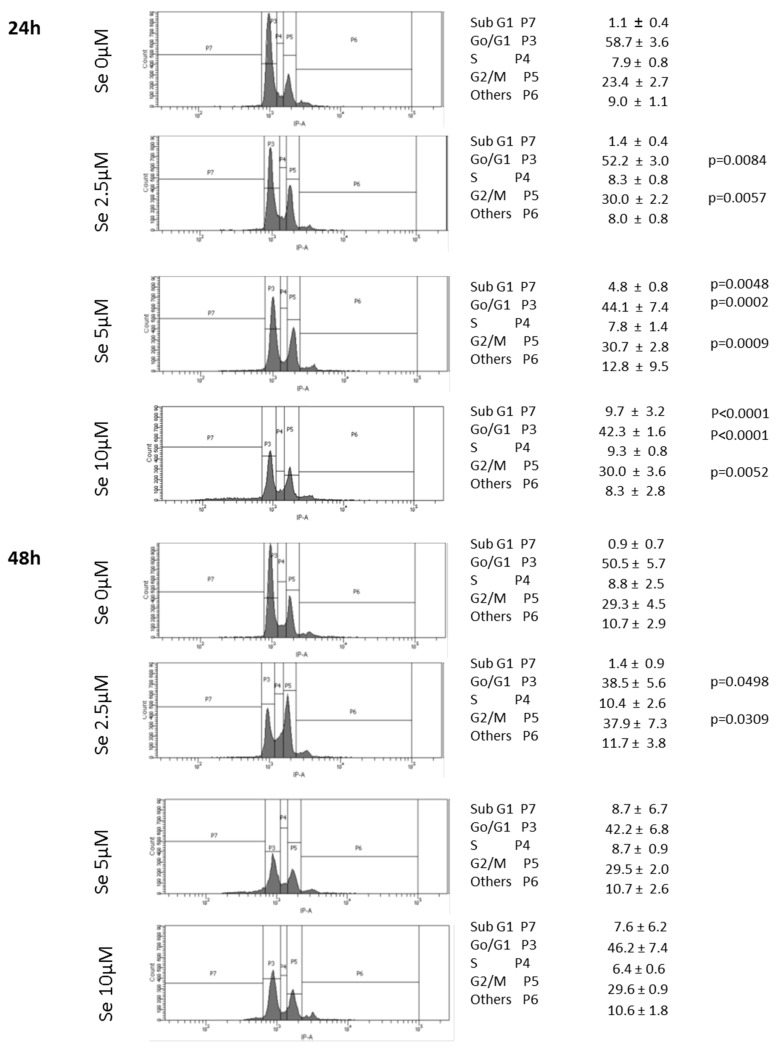
SS blocked the cell cycle in G2M. SS effects on R2J cell cycle (PI staining) was evaluated by flow cytometry analysis, 24 h and 48 h after SS treatment. Results are the mean ± SD of four independent experiments. Percentage of cells in each cell cycle phase was compared as a function of the SS concentration and when significant, the p value was informed. The subG1 was used to evaluate DNA fragmentation.

R2J-2D cell cycle was evaluated by flow cytometry after SS treatment. At 24 h-2.5 µM, SS significantly moved the cells from the G0/G1 to the G2M phase. This arrest in G2 was dose-dependent and was amplified at 48 h (Figure 6).

The subG0G1 analysis allowed evaluation of the DNA fragmentation, which was significantly induced by SS-24 h for the 5 and 10 µM treatment. For the higher doses used at 48 h, i.e., 5 and 10 µM, only a third and a quarter of the R2J cells remained, respectively, alive (Figure 5e), making the cell cycle analysis difficult.

### 2.11. SS Acted at the Epigenetic Level

**Figure 7:**
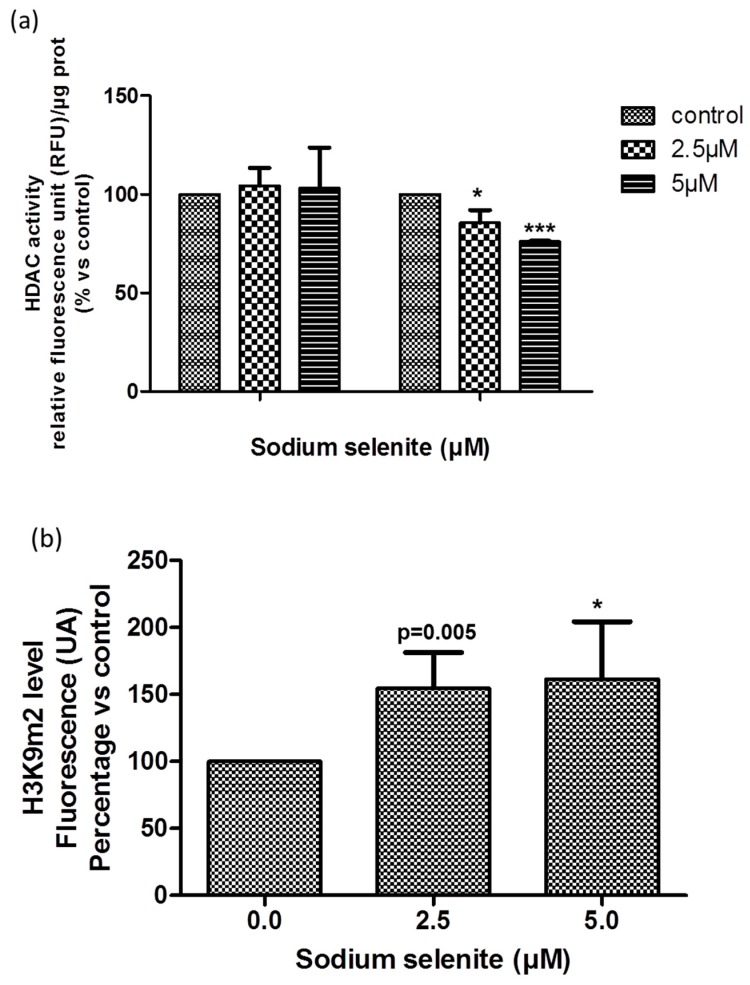
Epigenetic regulations under SS treatment. (**a**) SS altered HDAC activity. R2J-2D cells were not treated (control = 100%) or treated with 2.5 and 5 µM SS for 24 h, 48 h, and 72 h, and harvested. Cell lysates were obtained after five thaw-freeze cycles. HDAC activity (expressed in RFU) was determined using a fluorometric kit and results were normalized by the protein concentration in each sample. Results, expressed in percentage vs. control, are the mean ± SD of three independent experiments with * *p* < 0.05 and *** *p* < 0.0005 vs. control. (**b**) SS modulated the H3K9m2 level. 1 × 10^6^ R2J-2D cells fixed and permabilized were incubated with a monoclonal anti-dimethyl-H3K9 antibody to determine the methylation level of H3K9 after 24 h culture in the presence of SS at 2.5 and 5 µM versus not treated cells. A goat anti rabbit-FITC labelled antibody allowed the flow cytometric analyses with FACSCanto II. Results expressed in percentage vs. the control (not treated) are mean ± SD of five independent experiments, with * *p* < 0.05 vs. control.

HDAC activity was determined using a fluorometric analysis, after 24 h and 48 h of SS-treatment in R2J-2D cells. SS significantly altered the HDAC activity at 48 h-2.5 µM (Figure 7a).

H3K9m2 level was evaluated by flow cytometry after 24 h SS-treatment. SS treatment for 24 h increased H3K9m2 level in R2J cells as a tendency at 2.5 µM (*p* = 0.055) and significantly at 5 µM (*p* = 0.0318) (Figure 7b).

### 2.12. SS Induced Oxidative Stress, Autophagy, and Reticulum Endoplasmic Stress

**Figure 8:**
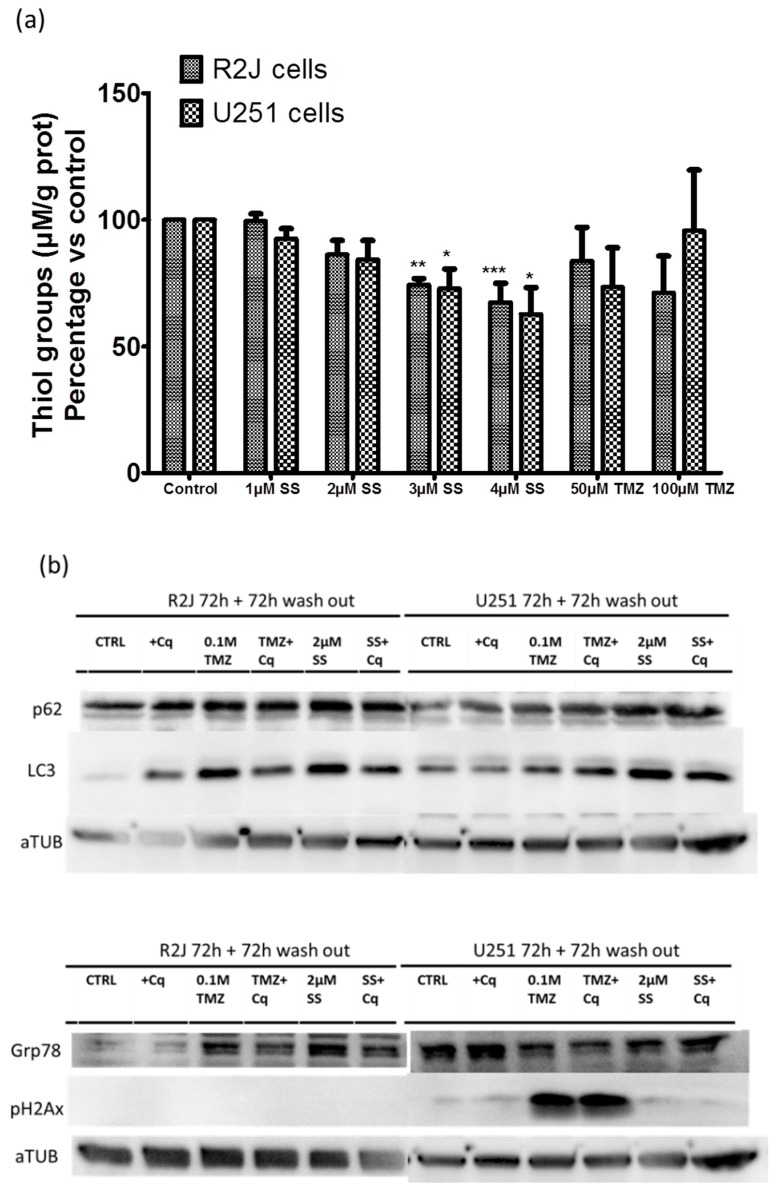
SS’s potential way of action was studied by evaluating (**a**) oxidative stress by thiol group levels in R2J and U251 cell lines after being treated for 24h with SS or TMZ. Cell lysates were obtained after five thaw-freeze cycles. Thiol groups (µM) were normalized by the protein concentration in each sample. Results, expressed in percentage vs. control (not treated cells), are the mean ± SD of three independent experiments with * *p* < 0.05, ** *p* < 0.005, and *** *p* < 0.0005 vs. control. (**b**) Autophagy (p62 and LC3-II), reticulum stress (Grp78), and DNA damage (pH2AX) by Western-blots analysis, 72 h after SS or TMZ treatments followed by 72 h of wash-out. Chloroquine (Cq) at 20 µM was used as a positive control to block autophagy 1 h before SS or TMZ treatments.

Oxidative stress was evaluated by quantifying thiol groups concentration in R2J and U251 cells, grown in the monolayer, after 24 h of SS treatment. SS significantly decreased thiol groups in both cell lines whereas TMZ did not (Figure 8a).

Western-blots showed that SS at 2 µM and TMZ at 100 µM repressed autophagy with an increase of p62 and LC3-II both in R2J and in U251 cells and induced a reticulum stress with an increase of Grp78 only in R2J cells (Figure 8b). It should be noted that chloroquine (Cq) at 20 µM was used as a blocker of autophagy. Interestingly, SS at 2 µM did not cause pH2AX expression (DNA damage) whereas TMZ did (Figure 8b). All these results suggest that SS and TMZ do not have the same mechanism of action.

## 3. Discussion

Despite the great efforts made to fight against GBM and the advent of TMZ as the first line CT agent available for long term application, the prognosis of patient suffering for GBM remains poor [1]. One problem is chemo-resistance, supposedly related to CSC, in which cells escape CT-induced cell death [33]. One hypothesis is that CSC can later differentiate into proliferative progenitor-like and more differentiated tumor cells that characterize the histological traits of the tumor entity [8]. New therapeutic approaches are likely to generate new drugs able to destroy the CSC of the tumor to stop the tumor progression.

In vitro tests are essential to raise these questions with the development of new cancer cell lines with CSC characteristics. Their individual phenotypic and/or genotypic characteristics provides a valuable means for this search [34,35,36]. The R2J cell line described herein was established from a CSF from a patient with a GBM. The karyotype and cell cycle analysis revealed a mixture of diploid (2n) and tetraploid (4n), but also 8n and 16n cells, a gain of chr-7 and -19, which may be due to an endoreplicative process frequently occurring in malignant cells and commonly associated to GBM [37].

There is a diversity of cellular phenotypes within the R2J-2D cell population that may reflect mixtures of progenitors, differentiated cells, and presumed cancer stem cells. Indeed, our data supports that R2J cells are derived from a tumor containing multipotent neural stem cell-like cells where a population of CSC persists. Indeed, R2J cells expressed Nestin and CD44, which is different from other usual reported GBM MGMTt+ cell lines, like T98G [13]. R2J cells are able to form spheres (1.9%) in a serum-free medium, with self-renewal capability, as reported for U87 (2.5%) [38] and U251 (1.15%) [39].

The most prevalent form of glioma is referred to as astrocytoma, based on the predominance of GFAP+ astrocytes-like cells within the tumor mass. GFAP is then used as an astrocytic lineage-specific marker [13]. The original tumor expressed GFAP, which was lost in adherent and R2J spheres. Furthermore, adherent R2J did not express CD56, a neuronal lineage marker, whereas the spheres expressed it, suggesting that R2J cells are more likely to differentiate into neurons than in astrocytes. Moreover, in R2J spheres, the strong expression of Nestin, Sox2, and CD44 also suggests that R2J cells are engaged in an incomplete GMT process that may induce a partial acquisition of some CSC markers. Indeed, recent studies have established a possible link between a passage through GMT and the acquisition of GSC properties. Thus, the expression of Nestin and Sox-2 has been associated with both the GMT process and CSC [8,9,10]. In mammary cells, the induction of EMT has also significantly led to the expression of markers associated with breast CSCs (for a review see [16]).

Proliferation may be altered by cellular signaling pathways modifications related to growth factor receptor expressions [2,3]. EGFR, associated to a poor prognosis [40], is expressed in some R2J cells, which are denoted with the polysomy of chr-7, but the abnormal karyotype of R2J cells is a common feature of GBM [41]. From all our results and on the basis of the molecular profiling established by Verhaak et al. [6,42] and on the new WHO classification [43], the R2J cell line could be classified into the mesenchymal-like subtype into the isocitrate deshydrogenase IDH-wildtype glioma subgroup. Additionally, the combination of a high expression of mesenchymal (CD44), progenitor cell markers (nestin, SOX2) is evocative of an epithelial (glial)-to-mesenchymal transition that has been linked to dedifferentiated and transdifferentiated tumors [17]. Moreover, the R2J cell line presents some similarities with the mesenchymal described USC02 cell line [44], with SOX2, CD44, and nestin expressions, and the capability to form spheres when cultured with growth factors, and to form tumors after intracranial implantation into nude mice.

The xenograft of R2J cells cultivated in 2D or in spheres resulted in the formation of a detectable tumor within 14 days post implantation into the striatum of nude mice. These results allowed conclusions on the tumorigenicity of this cell line and on its CSC properties [7]. Indeed, only 1000 spheres isolated from the total R2J-2D population were able to trigger a highly proliferative tumor growth.

As a first conclusion, although numerous GBM cell lines have been published, few of them have been established from tumor cells expressing MGMT transcript and having resistance to TMZ. Indeed, T98G is p53 mutation, has a positive MGMT status, and is resistant to TMZ [45]. Only U251 [39] and U87 [38] have been reported to contain a CSC population, but these are both MGMT negative. Moreover, LN229 and U251 are different from T98G and U87 by expressing nestin, SOX2, and CD44 [13]. Then, the R2J cell line really has added value because in addition to being MGMTt+, they exhibit CSC characteristics with in vivo tumorigenesis that are relevant to the testing of new drugs.

In this line, as we reported SS-anticancer effects in GBM cell lines cultivated in 2D [26] and in cell-cluster spheroids [27], we in vitro tested SS on the R2J-2D cells.

SS was cytotoxic in the micromolar range and much more toxic than TMZ, for which the IC50 was not reached at a higher dose and after long term treatment. This is consistent with the fact that the patient did not respond to TMZ that may be correlated with a MGMT transcript positive expression. Similar results were obtained in the T98G cell line, also positive for MGMT transcript expression [46], which exhibited a high TMZ resistance (IC50 was not reached at 1mM-72 h [27]).

R2J cells absorbed SS, and an uncomplete recovery suggest the generation of volatiles Se metabolites, as reported in prostate, colon, and Jurkat cell lines [47]. The SS absorption triggered DNA fragmentation and cell cycle arrest in G2, leading to both necrosis and apoptosis via caspase-3 activation, as reported in three GBM cell lines that were SS-treated [26]. These results are essential as R2J cells expressed a high level of HDAC that plays an important role in gene expression associated to cell proliferation (p21/Waf1, p27/kip1, p16/ink4a) and apoptosis via Bad, Trail, and FasL in glioma cells [48].

SS metabolism generated Se intermediates, which are also responsible for SS toxicity, such as selenide, selenocystine, and elemental Se [49]. Oxidative stress via the generation of anion superoxide is considered an essential process by which selenite applies its cytotoxicity [47,49].

To sustain an oxidative stress way of action and subsequent pathways, we showed that SS depleted thiol group concentration, in both R2J (p53 wild–type) and U251 (p53 mutant) cells, contrary to TMZ. Indeed, reactive oxygen species ROS production is dependent on the reduction of selenite to selenide through the consumption of thiols [50]. It was also shown that the changes in the redox status induced cell death via apoptosis and via mitochondria-selective autophagy (mitophagy) in glioma cells [29]. Western-blots experiments showed that SS repressed autophagy in both cell lines and induced reticulum stress only in R2J cells. Interestingly, SS at 2 µM did not cause double strand breaks whereas TMZ did. In parallel, TMZ did not deplete thiol groups, suggesting that SS and TMZ do not have the same mechanism of action and thus could have complementary effects to target GBM cells, if used in combination.

Epigenetic regulation of chromatin organization is a key regulator of gene expression that can significantly impact tumor biology and tumor response to therapies. In many tumor types, mutated or modified epigenetic regulators have been clearly associated to tumorigenesis because of their effects on gene expression (reviewed in [19]), and this has led to the development of pharmacologic inhibitors specific of these actors. At the epigenetic level, we focused on histone acetylation and methylation, both modulated by SS in R2J cells. Indeed, HDAC activity was significantly reduced by SS, as already reported in GBM cell lines [26]. In R2J cells, the inhibition of HDAC concomitant with cell proliferation braking and apoptosis induction is consistent with the role of HDAC in these two processes. Therefore, our results led to the intriguing question: How do both these epigenetic modifications interact and would they be able to re-express suppressor tumor genes? Some recent data have revealed an interplay between histone methylation, DNA methylation, the autophagy process, and apoptosis. An interesting study [51] showed that the histone methyltransferase, G9a, inhibits the expression of genes involved in the autophagy process. Indeed, the pharmacologic inhibition or genetic depletion of G9a triggers the formation of LC3B-II, the aggregation of p62, and the formation of autophagosomes. This loss of G9a leads to a reduction of H3K9m2 levels. In addition, a recent study [52] showed that BIX01294, an inhibitor of G9a, induced autophagy in glioma cells and stimulated differentiation of glioma stem cells (spheres cultivated without serum), associated with lower levels of H3K9m2 as the promoter of autophagy related genes. Taken together, our data suggest that SS could cause a regional remodeling of chromatin that may trigger apoptosis and cell cycle arrest.

## 4. Materials and Methods

### 4.1. Clinical Data of the Patient

In October 2009, the patient (a 49-year old white male without any medical history) felt faint and lost consciousness. Magnetic resonance imaging (MRI) showed a right tempo extensive process compatible with a GBM. In November 2009, the tumor was resected and histological analysis confirmed the diagnosis of GBM with a leptomeningeal extension (Figure 1). After the surgery, hydrocephalia and an unmanageable headache appeared. The patient was unfit to receive any conventional treatment, such as chemotherapy or/and radiotherapy.

Instead, the patient received weekly intrathecal injections of Trastuzumab because CSF glioma cells overexpressed HER2. In May 2010, symptoms and the leptomeningeal extension decreased, but the patient presented a local tumor progression on the MRI. So, the patient was treated with radiotherapy plus concomitant TMZ. In August 2010, because of tumor progression and leptomeningeal carcinomatosis progression, no more treatment was compatible with the patient’s health condition and he received standard palliative care. The patient died in October 2010.

### 4.2. Origin of R2J Cells

The tumor cells were isolated from the CSF of the patient (November 2009). The analysis of CSF showed numerous tumor cells with a high mitotic index, indicating a leptomeningeal carcinomatosis. These cells were cultured and the derived R2J cell line was successfully cryopreserved at different passages.

### 4.3. Morphological Analysis

The primary biopsy (original tumor) was processed for histopathologic study using standard methods and sections were stained with hematoxylin and eosin safran (HES).

R2J cells from the CSF were isolated by cytocentrifugation (Cytospin, Shandon, Pittsburgh, PA, USA) and stained using May Grunwald Giemsa (MGG) using a Zeiss microscope (Oberkochen, Germany). Pictures were taken using a Canon Power Shot S50 digital camera (Courbevoie, France).

### 4.4. Cell Culture

U251 cells were purchased from the American Type Culture Collection ATCC. They were cultured in DMEM medium containing 10% fetal calf serum (FCS) supplemented with penicillin (100 IU/mL), streptomycin (100 µg/mL, PS) and L-Glutamine (2mM) (Life Technology, Walthma, MA), in humidified normoxia incubator. U251 were harvested using 0.5%-Trypsin-EDTA (10×) (#15400-054, LifeTechnologies). U251 was chosen due to its MGMT negative and p53 mutant status.

R2J were cultured in RPMI 1640 medium enriched as described for U251, in humidified hypoxia incubator (3% O_2_, 5% CO_2_, 37 °C). R2J were harvested as described for U251.

For sphere formation, the R2J cells were grown in non-adherent 10-cm diameter Petri dishes (Greiner, les Ulis, France) coated with PolyHema (#P3932, Sigma, St Louis, MO, USA,) in DMEM-F12 medium (Life Technologies,) without serum containing 20 ng/mL human EGF, FGF-2, and 1× NeuroBrew-21 (Miltenyi Biotec, Bergirsch Gladbach, Germany), and PS (as above) in hypoxia. To conform with already published methods, the cells were plated at low density (10 per µL) to allow clonal sphere formation [53,54].

PolyHema was prepared at 10 mg/mL in ethanol 95% and sterilized by filtration on a Stericup (Millipore Express PLUS 1070, Temecula, CA, USA). To coat the 10 cm Petri-dish, 4mL of this solution was applied. The dishes were put in a dry incubator overnight, at 60 °C to dry the PolyHema.

### 4.5. Immunohistochemistry

Embedded paraffin tumor was analyzed in 2009 for CD56, Ki67, GFAP, NeuN, NF70, and synaptophysin, according to the current procedure of the laboratory. The Bouin’s fluid fixative used at this time is not compatible with the current methods of the laboratory and the immuno-histo staining obtained for some markers (IDH1 and olig2, for example) was unsatisfactory except for EGFR, CD44, CD34, and Nestin, performed after 2009.

R2J cells cultured in 2D or in spheres were fixed in formol 4%, 30 min before being included in HistogelTM (Microm Microtech, Francheville, France) and paraffin embedded. HES and IHC analyses were performed from 3 µm paraffin sections using a Histostain^®^ plus kit (Thermo Fisher Scientific, Waltham, MA) and Vector novaRED™ substrate kit for peroxidase or Ventana Benchmark^®^ XT platform (Roche, Rotkreug, Switzerland). Only nestin was revealed using Fast Red Bond Polymer Red Refine Detection (#DS93390, Leica Biosystems, Newcastle, UK).

CD-56 (#MAB24081) and Olig2 (#AF2418) antibodies are from R&D Systems (Temecula, CA, USA), MIB1/KI67 (#M7240), p53 (#M7001) and Vimentin (#M0725) were from Dako (Glostrup, Danemark), GFAP (#2202MGF, Eurodiagnostica, Malmö, Sweden), EGFR (#28-0005), E-Cadherin (#18-0223), and MDM2 (#33-7100) were from Zymed Lab, (South San Francisco, CA, USA), IDH1 (#DIA-H09, Histonova, Clinisciences, Nanterre, France), Nestin (#MAB5326), and NeuN (#MAB377) were from Zymed Lab (South San Francisco, CA, USA), ATRX (#HPA001906) and CD44 (#144M-95) were from Sigma Aldrich, P16 (#9511, MTM Roche, France), Neurofilament 70-200 (#Mob080, Diagnostic BioSystems, Pleasanton, CA, USA), and CD34 (#PN IM0786, Beckman Coulter, Brea, CA, USA).

### 4.6. Flow Cytometry Analysis

R2J-2D cells were seeded at 6000 cell/cm² in 10 cm Petri-dishes 48 h before treatment with SS (Sigma) for 24 h or 48 h, after which cells and medium were recovered, centrifuged (3 min, 360 g, RT), and cells were rinsed twice with PBS1X.

Apoptosis and the cell-cycle were evaluated with the FITC-Annexin V Apoptosis Detection kit and the Cycle Test Plus DNA reagent kit (BD Biosciences, San Jose, CA) following the manufacturer’s instructions. The analysis of cells in the subG1 phase was used to determine DNA fragmentation.

For dimethyl-Histone H3-Lysine-9 (H3K9m2) analysis, 10^6^ cells were fixed 30 min in PFA 1% on ice and then permeabilized with 0.1% Triton × 100 in PBS, 30 min on ice. R2J cells were washed in PBS/BSA 1% twice before adding 1 µL of the rabbit monoclonal antibody, anti-H3K9m2 (#MC554, Millipore), 45 min. The H3K9m2 level was revealed using a (FITC)-conjugated F(ab)′2 fragment goat anti-rabbit IgG(H+L) (Beckman Coulter), 30 min, 4 °C.

Cell fluorescence was detected with FACSCantoII (Becton Dickinson) and analysed with FACS Diva Software.

### 4.7. Chromosome Analysis

The chromosomes were analyzed according to the International System for Human Cytogenetic Nomenclature. Culture was exposed to 10 µg/mL colchicine for 60 min. Slides were processed for R-binding using heat and the Giemsa-RHG method [55].

### 4.8. Limiting Dilution/Tumor Sphere Initiation Assay (LDA)

An LDA was performed to quantify in R2J cell lines, the frequency of tumor spheres initiation cells (TS-ICs). R2J parental cells were calibrated to 1000 cells/mL (corresponding to 200 cells/200 µL/well) and then diluted into gradients of cell titers at 500, 250, 125, 62, 100, 50, 25, and 12.5 /mL and transferred into the wells of the 96-well microplate. Clonal spheres (non-adherent, tight, and spherical masses >5 cells) were counted under an inverted microscope (Leica, Wetzlar, Germany), at the end of two weeks. The results are expressed as the percentage of TS-ICs to examine the efficacy of clonogenesis [38].

### 4.9. Intracranial Cell Transplantation into Immunocompromised Mice

All animal protocols were approved by the GIN Animal Care and Use Committee (number 2017121517085709). R2J-2D cells (2 × 10^5^) or spheres (2 × 10^5^ or 1000) were implanted into the striatum of 4–6 week male athymicNudeFoxn1nu mice (*n* = 4, Envigo, Gannat, France) to analyze the tumor initiating capacity. Experimental details are available in Appendix A.

Mice were perfused; brains were removed and immediately fixed in formol 4% for subsequent histopathological analyses.

### 4.10. Magnetic Resonance Imaging

MRI sessions were performed at 4.7 T (Avance III console; Bruker, Ettlingen, Germany; IRMaGe MRI facility, Grenoble, France) using an actively decoupled cross-coil setup (volume coil for radiofrequency transmission and surface coil for signal reception). All MR experiments were performed under anaesthesia: 5% isoflurane for induction and 2% for maintenance in air. Mice were maintained at 37.0 °C and breath rate was cared for 60 breath/min throughout the acquisition. After shimming, MRI sequences were designed for anatomical T2-weighted (T2W) images acquisition using a spin-echo MRI sequence (TR /TE = 2500/33 ms, NA = 14, 21 slices with field of view (FOV) 20 × 20 mm^2^, matrix = 256 × 256 and voxel size = 156 × 156 × 500 μm^3^). Acquisition duration was 9 min 20 s. Tumor volume was calculated by adding each tumor surface × slice thickness.

### 4.11. Selenium Absorption

R2J-2D cells were seeded at 6000 cell/cm² in 10 cm Petri-dishes, 24 h before SS supplementation (2.5, 5, and 10 µM).

Se concentration was determined both in cell lysates and medium using an ICP-MS (X series II, Thermo Fisher Scientific, Waltham, Massachusetts, USA) equipped with Collision Cell interference elimination Technology (CCT) after dilution in 1% nitric acid containing Gallium (Ga) as an internal standard. The gas introduced in the CTT was a mix of He and H2. The isotope ratio ^78^Se/^71^Ga and an external calibration curve (16, 80, 160 nmol Se/L) allowed the determination of the Se concentration. The results (mean ± SD) were calculated in nmoles of Se per gram of protein and expressed as a percentage of the Se measured vs. Se added. The Se recovery was calculated by adding the quantity of Se both measured in the medium and in the lysates and compared to the Se quantity added (referred as 100%).

### 4.12. MTT Assay

A MTT (3-(4,5-Dimethylthiazol-2-yl)-2,5-Diphenyltetrazolium Bromide) assay (Sigma) was performed to determine R2J-2D cell survival after SS treatment at 2.5 to 12 µM for 24 h, 48 h, or 72 h. 1 × 10^5^ cells/well were seeded in 96-wells plate 24h before the addition of SS. The results (mean ± SD) are reported as the cell survival percentage versus the control (not treated). IC50 (concentrations inducing 50% cell death) values were determined by curve-fitting (using Prism 5, GraphPad).

In parallel, TMZ (Temodal, MSD, France) was dissolved in dimethyl sulfoxide (DMSO) and R2J cells were treated for 24 h, 48 h, and 72 h with 1, 10, 100, 500, and 1000 µM of TMZ. Cells were also treated with DMSO alone at the corresponding volume. A long term treatment was also performed by exposing R2J-2D cells to 400 µM TMZ for 72 h followed by a wash-out of 72 h. Results are expressed as the percentage of cell survival versus control (DMSO concentrations used were not cytotoxic).

### 4.13. Confocal Microscopy Analysis of Cell Viability

R2J-2D cells were set on a Lab-Tek-Chamber Slide system (Nalge Nunc International, Rochester, NY, USA) and were incubated with 5 µM of Fluorescein Diacetate (FDA, Thermofisher Scientific, Courtaboeuf, France), 1µM Hoechst 33342 (Interchim, Montluçon, France), and 10mg/mL Propidium Iodide (PI) (Interchim) in a 5% CO_2_ incubator, 37 °C, 20 min. After staining, cells were installed on the microscope incubation chamber with a controlled atmosphere at 37 °C and 5% CO_2_ (POC Chamber, Pecom, Erbach, Germany). Images were collected with a Leica TCS SP2 AOBS (Acoustico Optical Beam Splitter) inverted laser scanning confocal microscope equipped with an ×63 oil immersion objective (HCX PL APO 63.0× 1.40). Laser excitation and emission (adjusted with AOBS) were, respectively, 351–364/425–485 nm for Hoechst, 488/500–540 nm for FDA, and 543/600–650 nm for PI. Confocal pinhole (Airy units) was 1 for all channels. Each experiment was performed on a randomly chosen field. Raw image merging was obtained by LCS Lite software (Leica LCS, version 2.61, Overlay).

### 4.14. Caspase-3 Activity Evaluation

The caspase-3 Fluorometric Assay (BioVision, Mountain View, CA, USA) was used. R2J-2D cells were harvested and lysed into 50 μL of chilled Cell Lysis Buffer at 4 °C, 10 min, and centrifuged for 10 min, 10000 g at 4 °C. Protein concentration was determined (see below) and 50µL of supernatant containing 50 µg to 200 µg of protein was mixed with 50 µL of 2× reaction buffer and 5 μL of DEVD-AFC substrate (1 mM), in a 96-wells plate. Reaction was achieved for 1h at 37 °C. The plate was read on a fluorometer (Ex/Em = 400/505 nm). Caspase-3 activity is expressed in AU/g prot.

### 4.15. Determination of HDAC Activity

R2J-2D cells were treated for 24 h, 48 h, and 72 h with 2.5 and 5 µM SS and harvested. Pellets were rinsed twice in PBS and lysed in 400 µL sterile water, 10 min. HDAC activity was investigated in cell lysates from 50 µg total protein using a Fluorometric Assay (BioVision, Mountain View, CA, USA) as described by the manufacturer. HDAC activity is expressed in relative fluorescence units (RFU) per µg protein.

### 4.16. Real Time PCR

Total RNA extraction was performed with an RNeasy Mini Kit, following the manufacturer’s recommendations, with RNase-free DNase I treatment (Qiagen, Courtaboeuf, France). cDNA was reverse transcribed from 1 µg of total RNA with the SuperScriptIII First-Strand Synthesis (Life Technologies) followed by RNaseH (1 µL) treatment. Real time PCR was conducted using the QuantiTect SYBR Green RT-PCR kit (Qiagen) and the Stratagene 3005MxPro (Santa Clara, CA, USA). The primers (sequences in Supplementary Material Table S1) (Life Technologies) were all used at 400 nM, Tm at 60 °C, with the following qPCR program: 1 cycle: 15 min-95 °C, 40 cycles: 15 s-94 °C, 30 s-60 °C, 30 s-72 °C, 1 cycle: 1 min-95 °C, 30 s-60 °C, 30 s-95 °C.

Gene expression was quantified using the comparative threshold cycle (Ct) method [56] with a normalization against HPRT1, RPL27, and RPL32.

### 4.17. Thiol Group Determination

Thiol groups were determined by a colorimetric method using the reducing properties of the SH groups as previously described [25]. The thiol group levels were determined as micromoles per gram of total cell proteins and expressed in percentage versus the control.

### 4.18. Western Blot Analysis

U251 and R2J, after 72 h treatment followed by 72 h of wash-out with TMZ or SS or chloroquine (Cq, used to block autophagy 1 h before SS or TMZ treatment at 20 µM, Sanofi-Aventis), were harvested in protein extraction buffer (Tris-HCl 20 mM, pH 7.4, NaCl 137 mM, EDTA 2 mM, Triton X-100 1%, supplemented with proteases’ and phosphatases’ inhibitors (1/100, Sigma-Aldrich). After pipetting up and down with a P1000, supernatants were collected after centrifugation at 10,000 g for 10 min, at 4 °C. Protein concentration was measured by the BCA method. Proteins (20 µg total) were treated with SDS-PAGE sample buffer [6× concentrated: 350 mM Tris, 10% (w/v) SDS, 30% (v/v) glycerol, 0.6 M DTT, 0.06% (w/v) bromophenol blue], boiled for 5 min at 95 °C, and loaded on 15% (for LC3, p62, and phospho-H2Ax) and 8% (for Grp78) acrylamide/bisacrylamide gels. Proteins were transferred onto nitrocellulose membranes, blocked with 5% bovine serum albumin containing 0.5% Tween (Euromedex) (PBST) for 1 h at room temperature, and probed overnight at 4 °C with the following primary antibodies diluted in 5% bovine serum albumin-PBST: 1:1000 rabbit anti-p62 antibody (Sigma-Aldrich), 1:200 goat anti-Grp78 antibody (Santa Cruz Biotechnology, Dallas, TX), 1:1000 anti-LC3-II antibody, 1:1000 rabbit anti-phospho-H2Ax antibody (both from Cell Signalling Technology), and 1:5000 mouse anti-alpha tubulin (Sigma-Aldrich). Membranes were washed with PBST, incubated for 1 h at room temperature with horseradish peroxidase-conjugated secondary antibodies (from Jackson Immunoresearch), and visualized using electrochemiluminescence (ECL) western blotting substrate (ThermoFisher Scientific) on a Chemidoc imaging system (BioRad, Hercules, CA).

### 4.19. Protein Concentration Determination

The concentration of protein in cell lysates (obtained as mentioned above) was determined using the BCA assay (Interchim, Montluçon, France) as previously described [24]. The absorption at 562 nm was measured using the Varioskan Flash (Thermo Fisher Scientific) associated to the SkanIt Software for the quantification.

### 4.20. Statistical Analysis

Results are expressed as means ± SD for the number of experiments indicated. All statistical analysis of data was computed using StatView^®^ (SAS Institute, CA, USA). The sources of variation for multiple comparisons were assessed by analysis of variance ANOVA. The differences were considered statistically significant at *p* < 0.05 versus the control.

## 5. Conclusions

In conclusion, we described a newly established glioblastoma cell line (R2J), from a CSF from a patient affected by a metastatic GBM. R2J were MGMT+ and resistant to TMZ. In the presence of FGFb and EGF, the R2J forming spheres were phenocopied on 2D cultured R2J cells except for CD56, a neuronal marker, suggesting that R2J may differentiate into neuronal-like cells. The cell line could form a tumor when intracranially implanted into nude mice even at 1000 spheres, providing conclusions on their CSC properties. In vitro, SS could exert its anti-cancer properties in R2J cells, and is thus relevant for testing in this new GBM bearing mouse model.

## Figures and Tables

**Table 1:** Immunohistochemical characterisation of the original tumor compared to R2J monolayer cells and to R2J forming spheres. The expression level is expressed as followed: -: no expression, +: positive expression, nd: Not determined.

Related Family	Proteins	Tumor	R2J Monolayer (2D)	R2J-Gliospheres
Conventional GBM markers and subtypes	ATRX	nd	+	+
	GFAP	+	−	−
	IDH1	nd	−	−
	Ki67	+	+	+
	Olig2	−	−	+ rare cells
	TP53 mutated	nd	−	−
	Vimentin	+	+	+
Neuronal markers	CD56	+	−	+
	neuN	−	−	−
	NF70	−	−	−
Growth factor receptors	EGFR	+ low expression	+ few cells	+ few cells
Cell cycle markers	P16/INK4	nd	+	+
	MDM2	nd	−	−
Cancer stem cell markers	CD34	−	−	−
	Nestin	+	+	+
Mesenchymal shift	CD44	+ rare cells	+ (>90%)	+ few cells
	E-cadherin	nd	−	−

## Supplementary Materials

The following are available online at http://www.mdpi.com/2072-6694/11/1/12/s1, Figure S1: A representative G-banded karyotype of R2J cells. The R2J cell line was identified as near-triploid (hypotriploid) with 64 chromosomes, Table S1: primer sequences used for RT-q-PCR.

## Abbreviations

2D	Monolayer
BSA	Bovine Serum Albumin
CSC	Cancer Stem Cell
CSF	Cerebro-Spinal Fluid
Ct	Cycle threshold
CT	ChemoTherapy
ECM	ExtraCellular Matrix
EGFR	Epithelial Growth Factor-Receptor
GMT	Glial to Mesenchymal Transition
FACS	Fluorescence Activated Cell Sorter
FCS	Foetal Calf Serum
FDA	Fluorescein DiAcetate
FISH	Fluorescence In Situ Hybridization
FITC	Fluorescein IsoThioCyanate
G-CSF-R	Granulocyte-Colony Stimulating Factor-Receptor
GFAP	Glial Fibrillary Acidic Protein
H3K9m2	dimethyl-Histone-3-lysine-9
HDAC	Histone Deacetylase
HES	Hematoxylin Eosin Safran
ICP-MS	Inductively Coupled Plasma Mass Spectrometry
IDH	Isocitrate DesHydrogenase
KDM	Histone Lysine Demethylase
MDM2	Mouse Double Minute 2 homolog
MGG	May Grunwald Giemsa
MGMTt+	positive transcript expression of O(6)-MethyGuanine-DNA-MethylTransferase
MMPs	Matrix Metallo-Proteases
MRI	Magnetic Resonance Imaging
N-CAM	Neural Cell Adhesion Molecule
neuN	NEUronal Nuclear antigen
p15/INK4B	Inhibitor of CDK4B
p16/INK4A	Inhibitor of CDK4A
p21/WAF1	Cyclin-dependent kinase inhibitor 1
p27/KIP1	Cyclin-dependent kinase inhibitor 1B
PBS	Phosphate Buffer Saline
PI	Propidium iodide
PI3K	Phosphatidylinositol-3 kinase
PBS	Phosphate buffer salt
PFA	ParaFormAldehyde
ROS	Reactive Oxygen Species
RT	Radiation Therapy
TMZ	Temozolomide
SS	Sodium selenite

## Appendix A

Cells were harvested using Accutase (Sigma), counted, and suspended in 10 mL PBS. Cell viability and counting were assessed before and after the procedure using Scepter^®^ (Millipore). All surgery was performed using sterile techniques within a tissue culture hood. Mice were anesthetized with isoflurane 5% and maintained under 2.5% during the surgery. NaCl 0.9% was put on eyes throughout the procedure and mice were kept on a warm pad throughout. Cells were injected into the right striatum using a stereotaxic apparatus. Injection coordinates were 2.6 mm lateral to Bregma and a 3.6mm depth. A small bore hole was made using a 25 g needle. The cell suspension was mixed and loaded into the syringe just prior to injection. 3 µL of cells was delivered using a 26 g Hamilton microsyringe (701RN, 10 µL). The syringe was stabilized for 1 min before injection and 0.5 µL of cell suspension was injected each 30 s. The needle was kept in place for a further 4 min to prevent backflow and was then removed slowly (1 µL/min). After cell injection, the skin incisions were closed. To avoid suffering, Buprecare^®^ was intracutaneously injected (0.1 mg/kg) to each mouse. The animals were then revived under an infrared lamp and then returned with their congeners.

## References

[1] Stupp R., Hegi M.E., Mason W.P., van den Bent M.J., Taphoorn M.J., Janzer R.C., Ludwin S.K., Allgeier A., Fisher B., Belanger K. (2009). Effects of radiotherapy with concomitant and adjuvant temozolomide versus radiotherapy alone on survival in glioblastoma in a randomised phase III study: 5-year analysis of the EORTC-NCIC trial. Lancet Oncol..

[2] Vivanco I., Sawyers C.L. (2002). The phosphatidylinositol 3-Kinase AKT pathway in human cancer. Nat. Rev. Cancer.

[3] Vermeulen K., Van Bockstaele D.R., Berneman Z.N. (2003). The cell cycle: A review of regulation, deregulation and therapeutic targets in cancer. Cell Prolif..

[4] Bonomi S., Gallo S., Catillo M., Pignataro D., Biamonti G., Ghigna C. (2013). Oncogenic alternative splicing switches: Role in cancer progression and prospects for therapy. Int. J. Cell Biol..

[5] Mao X., Hamoudi R.A. (2000). Molecular and cytogenetic analysis of glioblastoma multiforme. Cancer Genet. Cytogenet..

[6] Ceccarelli M., Barthel F.P., Malta T.M., Sabedot T.S., Salama S.R., Murray B.A., Morozova O., Newton Y., Radenbaugh A., Pagnotta S.M. (2016). Molecular Profiling Reveals Biologically Discrete Subsets and Pathways of Progression in Diffuse Glioma. Cell.

[7] Chen J., Li Y., Yu T.S., McKay R.M., Burns D.K., Kernie S.G., Parada L.F. (2012). A restricted cell population propagates glioblastoma growth after chemotherapy. Nature.

[8] Yu Z., Pestell T.G., Lisanti M.P., Pestell R.G. (2012). Cancer stem cells. Int. J. Biochem. Cell Biol..

[9] Jin X., Jin X., Jung J.E., Beck S., Kim H. (2013). Cell surface Nestin is a biomarker for glioma stem cells. Biochem. Biophys. Res. Commun..

[10] Mimeault M., Batra S.K. (2013). Molecular biomarkers of cancer stem/progenitor cells associated with progression, metastases, and treatment resistance of aggressive cancers. Cancer Epidemiol. Prev. Biomark..

[11] Choi S.A., Wang K.C., Phi J.H., Lee J.Y., Park C.K., Park S.H., Kim S.K. (2012). A distinct subpopulation within CD133 positive brain tumor cells shares characteristics with endothelial progenitor cells. Cancer Lett..

[12] Singh S.K., Clarke I.D., Terasaki M., Bonn V.E., Hawkins C., Squire J., Dirks P.B. (2003). Identification of a cancer stem cell in human brain tumors. Cancer Res..

[13] Hong X., Chedid K., Kalkanis S.N. (2012). Glioblastoma cell line-derived spheres in serumcontaining medium versus serum-free medium: A comparison of cancer stem cell properties. Int. J. Oncol..

[14] Thiery J.P., Sleeman J.P. (2006). Complex networks orchestrate epithelial-mesenchymal transitions. Nat. Rev. Mol. Cell Biol..

[15] Mahabir R., Tanino M., Elmansuri A., Wang L., Kimura T., Itoh T., Ohba Y., Nishihara H., Shirato H., Tsuda M. (2013). Sustained elevation of Snail promotes glial-mesenchymal transition after irradiation in malignant glioma. Neuro Oncol..

[16] May C.D., Sphyris N., Evans K.W., Werden S.J., Guo W., Mani S.A. (2011). Epithelial-mesenchymal transition and cancer stem cells: A dangerously dynamic duo in breast cancer progression. Breast Cancer Res..

[17] Singh A., Settleman J. (2010). EMT, cancer stem cells and drug resistance: An emerging axis of evil in the war on cancer. Oncogene.

[18] Harabin-Slowinska M., Slowinski J., Konecki J., Mrowka R. (1998). Expression of adhesion molecule CD44 in metastatic brain tumors. Folia Neuropathol..

[19] Wilting R.H., Dannenberg J.H. (2012). Epigenetic mechanisms in tumorigenesis, tumor cell heterogeneity and drug resistance. Drug Resist. Updat..

[20] Seligson D.B., Horvath S., McBrian M.A., Mah V., Yu H., Tze S., Wang Q., Chia D., Goodglick L., Kurdistani S.K. (2009). Global levels of histone modifications predict prognosis in different cancers. Am. J. Pathol..

[21] Chen M.W., Hua K.T., Kao H.J., Chi C.C., Wei L.H., Johansson G., Shiah S.G., Chen P.S., Jeng Y.M., Cheng T.Y. (2010). H3K9 histone methyltransferase G9a promotes lung cancer invasion and metastasis by silencing the cell adhesion molecule Ep-CAM. Cancer Res..

[22] Zang L., Kondengaden S.M., Che F., Wang L., Heng X. (2018). Potential Epigenetic-Based Therapeutic Targets for Glioma. Front. Mol. Neurosci..

[23] Park C.K., Lee S.H., Kim T.M., Choi S.H., Park S.H., Heo D.S., Kim I.H., Jung H.W. (2013). The value of temozolomide in combination with radiotherapy during standard treatment for newly diagnosed glioblastoma. J. Neurooncol..

[24] Hazane-Puch F., Champelovier P., Arnaud J., Garrel C., Ballester B., Faure P., Laporte F. (2013). Long-term selenium supplementation in HaCaT cells: Importance of chemical form for antagonist (protective versus toxic) activities. Biol. Trace Elem. Res..

[25] Hazane-Puch F., Champelovier P., Arnaud J., Trocme C., Garrel C., Faure P., Laporte F. (2014). Six-day selenium supplementation led to either UVA-photoprotection or toxic effects in human fibroblasts depending on the chemical form and dose of Se. Met. Integr. Biomet. Sci..

[26] Hazane-Puch F., Arnaud J., Trocme C., Faure P., Laporte F., Champelovier P. (2016). Sodium Selenite Decreased HDAC Activity, Cell Proliferation and Induced Apoptosis in Three Human Glioblastoma Cells. Anti-Cancer Agents Med. Chem..

[27] Berthier S., Arnaud J., Champelovier P., Col E., Garrel C., Cottet C., Boutonnat J., Laporte F., Faure P., Hazane-Puch F. (2017). Anticancer properties of sodium selenite in human glioblastoma cell cluster spheroids. J. Trace Elem. Med. Biol..

[28] Rooprai H.K., Kyriazis I., Nuttall R.K., Edwards D.R., Zicha D., Aubyn D., Davies D., Gullan R., Pilkington G.J. (2007). Inhibition of invasion and induction of apoptosis by selenium in human malignant brain tumour cells in vitro. Int. J. Oncol..

[29] Kim E.H., Sohn S., Kwon H.J., Kim S.U., Kim M.J., Lee S.J., Choi K.S. (2007). Sodium selenite induces superoxide-mediated mitochondrial damage and subsequent autophagic cell death in malignant glioma cells. Cancer Res..

[30] Zhang Z., Chinen Y., Zhu Z., Kimura M., Itokawa Y. (1995). Uptake and distribution of sodium selenite in rat brain tumor. Biol. Trace Elem. Res..

[31] Cavalieri R.R., Scott K.G., Sairenji E. (1966). Selenite (75Se) as a tumor-localizing agent in man. J. Nucl. Med..

[32] Hazane-Puch F., Soldini A. (2017). Unit Nutritional and Hormonal Biochemistry, Institute of Biology and Pathology, Grenoble Alpes Hospital, CS10217, 38043 Grenoble Cedex 9, France.

[33] Beier D., Schulz J.B., Beier C.P. (2011). Chemoresistance of glioblastoma cancer stem cells—Much more complex than expected. Mol. Cancer.

[34] Loja T., Chlapek P., Kuglik P., Pesakova M., Oltova A., Cejpek P., Veselska R. (2009). Characterization of a GM7 glioblastoma cell line showing CD133 positivity and both cytoplasmic and nuclear localization of nestin. Oncol. Rep..

[35] Onda K., Nagai S., Tanaka R., Morii K., Yoshimura J.I., Tsumanuma I., Kumanishi T. (1999). Establishment of two glioma cell lines from two surgical specimens obtained at different times from the same individual. J. Neuro-Oncol..

[36] Pollard S.M., Yoshikawa K., Clarke I.D., Danovi D., Stricker S., Russell R., Bayani J., Head R., Lee M., Bernstein M. (2009). Glioma stem cell lines expanded in adherent culture have tumor-specific phenotypes and are suitable for chemical and genetic screens. Cell Stem Cell.

[37] Therman E., Buchler D.A., Nieminen U., Timonen S. (1984). Mitotic modifications and aberrations in human cervical cancer. Cancer Genet. Cytogenet..

[38] Yu S.C., Ping Y.F., Yi L., Zhou Z.H., Chen J.H., Yao X.H., Gao L., Wang J.M., Bian X.W. (2008). Isolation and characterization of cancer stem cells from a human glioblastoma cell line U87. Cancer Lett..

[39] Iacopino F., Angelucci C., Piacentini R., Biamonte F., Mangiola A., Maira G., Grassi C., Sica G. (2014). Isolation of cancer stem cells from three human glioblastoma cell lines: Characterization of two selected clones. PLoS ONE.

[40] Kong D.S., Song S.Y., Kim D.H., Joo K.M., Yoo J.S., Koh J.S., Dong S.M., Suh Y.L., Lee J.I., Park K. (2009). Prognostic significance of c-Met expression in glioblastomas. Cancer.

[41] Westphal M., Hansel M., Hamel W., Kunzmann R., Holzel F. (1994). Karyotype analyses of 20 human glioma cell lines. Acta Neurochir..

[42] Verhaak R.G., Hoadley K.A., Purdom E., Wang V., Qi Y., Wilkerson M.D., Miller C.R., Ding L., Golub T., Mesirov J.P. (2010). Integrated genomic analysis identifies clinically relevant subtypes of glioblastoma characterized by abnormalities in PDGFRA, IDH1, EGFR, and NF1. Cancer Cell.

[43] Louis D.N., Perry A., Reifenberger G., von Deimling A., Figarella-Branger D., Cavenee W.K., Ohgaki H., Wiestler O.D., Kleihues P., Ellison D.W. (2016). The 2016 World Health Organization Classification of Tumors of the Central Nervous System: A summary. Acta Neuropathol..

[44] Jhaveri N., Agasse F., Armstrong D., Peng L., Commins D., Wang W., Rosenstein-Sisson R., Vaikari V.P., Santiago S.V., Santos T. (2016). A novel drug conjugate, NEO212, targeting proneural and mesenchymal subtypes of patient-derived glioma cancer stem cells. Cancer Lett..

[45] Lee S.Y. (2016). Temozolomide resistance in glioblastoma multiforme. Genes Dis..

[46] Okamoto R., Takano H., Okamura T., Park J.S., Tanimoto K., Sekikawa T., Yamamoto W., Sparreboom A., Verweij J., Nishiyama M. (2002). O(6)-methylguanine-DNA methyltransferase (MGMT) as a determinant of resistance to camptothecin derivatives. Jpn. J. Cancer Res..

[47] Lunoe K., Gabel-Jensen C., Sturup S., Andresen L., Skov S., Gammelgaard B. (2011). Investigation of the selenium metabolism in cancer cell lines. Met. Integr. Biomet. Sci..

[48] Yin D., Ong J.M., Hu J., Desmond J.C., Kawamata N., Konda B.M., Black K.L., Koeffler H.P. (2007). Suberoylanilide hydroxamic acid, a histone deacetylase inhibitor: Effects on gene expression and growth of glioma cells in vitro and in vivo. Clin. Cancer Res..

[49] Weekley C.M., Aitken J.B., Vogt S., Finney L.A., Paterson D.J., de Jonge M.D., Howard D.L., Witting P.K., Musgrave I.F., Harris H.H. (2011). Metabolism of selenite in human lung cancer cells: X-ray absorption and fluorescence studies. J. Am. Chem. Soc..

[50] Olm E., Fernandes A.P., Hebert C., Rundlof A.K., Larsen E.H., Danielsson O., Bjornstedt M. (2009). Extracellular thiol-assisted selenium uptake dependent on the x(c)-cystine transporter explains the cancer-specific cytotoxicity of selenite. Proc. Natl. Acad. Sci. USA.

[51] Artal-Martinez de Narvajas A., Gomez T.S., Zhang J.S., Mann A.O., Taoda Y., Gorman J.A., Herreros-Villanueva M., Gress T.M., Ellenrieder V., Bujanda L. (2013). Epigenetic regulation of autophagy by the methyltransferase G9a. Mol. Cell Biol..

[52] Ciechomska I.A., Przanowski P., Jackl J., Wojtas B., Kaminska B. (2016). BIX01294, an inhibitor of histone methyltransferase, induces autophagy-dependent differentiation of glioma stem-like cells. Sci. Rep..

[53] Coles-Takabe B.L., Brain I., Purpura K.A., Karpowicz P., Zandstra P.W., Morshead C.M., van der Kooy D. (2008). Don’t look: Growing clonal versus nonclonal neural stem cell colonies. Stem Cells.

[54] Singh S.K., Hawkins C., Clarke I.D., Squire J.A., Bayani J., Hide T., Henkelman R.M., Cusimano M.D., Dirks P.B. (2004). Identification of human brain tumour initiating cells. Nature.

[55] Sehested J. (1974). A simple method for R banding of human chromosomes, showing a pH-dependent connection between R and G bands. Humangenetik.

[56] Giulietti A., Overbergh L., Valckx D., Decallonne B., Bouillon R., Mathieu C. (2001). An overview of real-time quantitative PCR: Applications to quantify cytokine gene expression. Methods.

