# Supplementary material for: A New Patient-Derived Metastatic Glioblastoma Cell Line: Characterisation and Response to Sodium Selenite Anticancer Agent
Source: Cancers (Basel). 2018 Dec 21;11(1):12. doi: 10.3390/cancers11010012 (PMC6356827; doi:10.3390/cancers11010012)
Supplement: Supplementary file 1 [file cancers-11-00012-s001.pdf]

cancers

Supplementary Materials: A New Patient-Derived Metastatic Glioblastoma Cell Line: Characterisation and Response to Sodium Selenite Anticancer Agent

Sylvie Berthier, Louis Larrouquère, Pierre Champelovier, Edwige Col, Christine Lefebvre, Cécile Cottet-Rouselle, Josiane Arnaud, Catherine Garrel, François Laporte, Jean Boutonnat, Patrice Faure and Florence Hazane-Puch

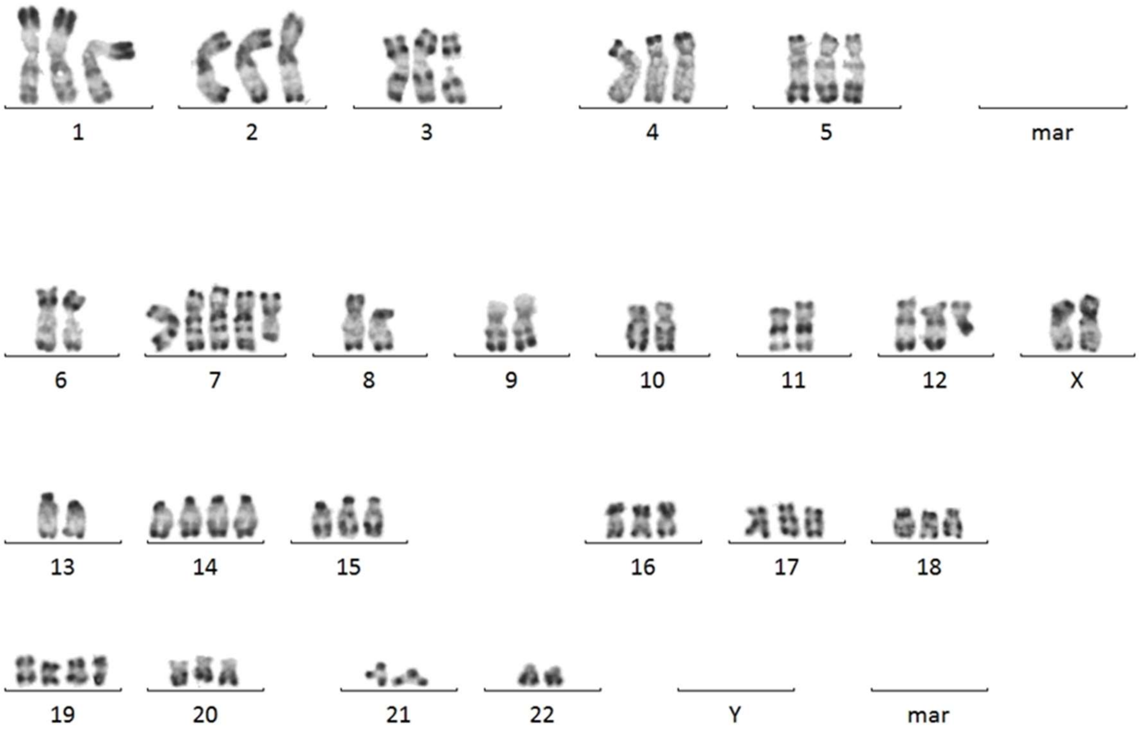

Figure S1. A representative G-banded karyotype of R2J cells. The R2J cell line was identified as near-triploid (hypotriploid) with 64 chromosomes.

Table S1. primer sequences used for RT-q-PCR.

Name	Forward (5′–3′)	Reverse (5′–3′)	Product Size (pb)
E-Cadherin	ATTTTTCCCTCGACACCCGAT	TCCCAGGCGTAGACCAAGA	109
HPRT1	CTCATGGACTAATTATGGACAGGAC	GCAGGTCAGCAAAGAATTTATAGCC	123
MGMT	ACCGTTTGCGACTTGGTACTT	GGAGCTTTATTTCGTGCAGACC	127
N-Cadherin	TCAGGCTGTGGACATAGAAACC-	GCTGTAAACGACTCTGGCACT	217
RPL27	TGATGGCACCTCAGATCGC	AGAGTACCTTGTGGGCATTAGG	240
RPL32	TTAAGCGTAACTGGCGGAAAC	GAGCGATCTCGGCACAGTAA	210
SOX2	TGGACAGTTACGCGCACAT	CGAGTAGGACATGCTGTAGGT	215
